# Adding New Scientific Evidences on the Pharmaceutical Properties of *Pelargonium quercetorum* Agnew Extracts by Using In Vitro and In Silico Approaches

**DOI:** 10.3390/plants12051132

**Published:** 2023-03-02

**Authors:** Annalisa Chiavaroli, Maria Loreta Libero, Simonetta Cristina Di Simone, Alessandra Acquaviva, Nilofar, Lucia Recinella, Sheila Leone, Luigi Brunetti, Donatella Cicia, Angelo Antonio Izzo, Giustino Orlando, Gokhan Zengin, Abdullahi Ibrahim Uba, Ugur Cakilcioğlu, Muzaffer Mukemre, Omer Elkiran, Luigi Menghini, Claudio Ferrante

**Affiliations:** 1 Department of Pharmacy, “G. d’Annunzio” University of Chieti-Pescara, 66100 Chieti, Italy; 2 Department of Pharmacy, School of Medicine, University of Naples Federico II, 80131 Naples, Italy; 3 Physiology and Biochemistry Laboratory, Department of Biology, Science Faculty, Selcuk University, 42130 Konya, Turkey; 4 Department of Molecular Biology and Genetics, Istanbul AREL University, 34537 Istanbul, Turkey; 5 Pertek Sakine Genç Vocational School, Munzur University, 62500 Pertek, Turkey; 6 Department of Plant and Animal Production, Yuksekova Vocational School, Hakkari University, 30100 Hakkari, Turkey; 7 Department of Environmental Health, Vocational School of Health Services, Sinop University, 57000 Sinop, Turkey

**Keywords:** *Pelargonium quercetorum*, phenolic compounds, antioxidant, enzyme inhibition, colon inflammation, TRPM8, COX-2, TNFα

## Abstract

*Pelargonium quercetorum* is a medicinal plant traditionally used for treating intestinal worms. In the present study, the chemical composition and bio-pharmacological properties of *P. quercetorum* extracts were investigated. Enzyme inhibition and scavenging/reducing properties of water, methanol, and ethyl acetate extracts were assayed. The extracts were also studied in an ex vivo experimental model of colon inflammation, and in this context the gene expression of cyclooxygenase-2 (COX-2) and tumor necrosis factor α (TNFα) were assayed. Additionally, in colon cancer HCT116 cells, the gene expression of transient receptor potential cation channel subfamily M (melastatin) member 8 (TRPM8), possibly involved in colon carcinogenesis, was conducted as well. The extracts showed a different qualitative and quantitative content of phytochemicals, with water and methanol extracts being richer in total phenols and flavonoids, among which are flavonol glycosides and hydroxycinnamic acids. This could explain, at least in part, the higher antioxidant effects shown by methanol and water extracts, compared with ethyl acetate extract. By contrast, the ethyl acetate was more effective as cytotoxic agent against colon cancer cells, and this could be related, albeit partially, to the content of thymol and to its putative ability to downregulate TRPM8 gene expression. Additionally, the ethyl acetate extract was effective in inhibiting the gene expression of COX-2 and TNFα in isolated colon tissue exposed to LPS. Overall, the present results support future studies for investigating protective effects against gut inflammatory diseases.

## 1. Introduction

*Pelargonium quercetorum* Agnew, belonging to the Gerianaceae family, is a medicinal plant traditionally used by the local population in the Hakkari region of Turkey and in the Kurdistan region of Iraq [1]. The species is characterized by herbaceous or semi-woody shrub plants, with rhizomes, schizocarpic fruits, and orange-red or pink flowers [2]. In Turkey, it is used for treating sore throats and as a wound healing agent, as well as for the management of migraines and pain [1]. The aerial portion is used as antiparasitic; indeed, a recent paper demonstrated the antiparasitic properties of the water extract against *Leishmania major* [3]. The methanol extract of the whole plant was reported to induce a cytotoxic activity against lung cancer cells [4], and was also effective as anti-inflammatory agent, in macrophages challenged with *Escherichia coli* lipopolysaccharide (LPS) [5]. Similarly, other *Pelargonium* species, especially *P. sidoides*, have been used for the treatment of gastrointestinal and liver diseases [6], but also for treating respiratory disorders [7].

Regarding the chemical composition, *Pelargonium* species are rich in essential oils, mainly constituted by monoterpenes, which can play antimicrobial and cytotoxic activities [4]. In the methanol extract of *P. quercetorum* the hydrocarbons tetracosane, heneicosane, and 2-methyleicosane were the phytochemicals present in higher concentration [4]; whereas rutin and shikimic acid were reported to be the main phenolics present in the methanol extract [8]. Ferda et al. [9] described cytotoxic and pro-apoptotic effects by *P. quercetorum*, and scavenging/reducing properties that could be partially related to the total phenol content of the methanol extract.

In the present study, the chemical composition and biological properties of different polarity extracts of *P. quercetorum* were investigated. Specifically, phenolic compounds were assayed through colorimetric and chromatographic analysis. Intrinsic enzyme inhibition (against α-glucosidase, α-amylase, cholinesterases, and tyrosinase) and scavenging/reducing properties were assayed as well. The extracts were also studied in an ex vivo experimental model of colon inflammation, constituted by isolated colon specimens exposed to LPS, in order to reproduce the burden of inflammation and oxidative stress occurring during colitis, in vivo [10,11,12,13]. In this context, the gene expression of pro-inflammatory biomarkers, including cyclooxygenase-2 (COX-2) and tumor necrosis factor α (TNFα) were assayed. Additionally, the gene expression of vascular endothelial growth factor A, hypoxia-inducible factor 1α (HIF-1α), deeply involved in the so-called inflammatory-to-cancer transition [14], was also measured in human colon cancer HCT116 cells. In the same cell model, the gene expression of transient receptor potential cation channel subfamily M (melastatin) member 8 (TRPM8), possibly involved in colon carcinogenesis [15,16], was conducted as well.

Finally, in silico studies were carried out for predicting the pharmacokinetics of the main phytochemicals present in the extracts, as well as direct interactions with protein targets assayed through the abovementioned biological models, among which the enzymes tyrosinase, α-amylase, α-glucosidase, cholinesterases, and the TRPM8 receptor.

## 2. Results and Discussion

### 2.1. Total Bioactive Compounds

Phenolic compounds are considered to be one of the most important players among the specialized metabolites and therefore the determination of their content is an important parameter to evaluate the pharmacological potential of plant extracts. In the present study, the total contents of phenolics and flavonoids were determined, and the results are summarized in Table 1. The highest total phenolic content (TPC) was found in the water extract with 62.52 mg GAE/g extract, followed by methanol (45.77 mg GAE/g extract), and ethyl acetate (21.94 mg GAE/g extract). For total flavonoid content (TFC), the order was methanol (40.12 mg RE/g extract), water (16.15 mg RE/g extract), and ethyl acetate (2.78 mg RE/g extract). In a previous study by Karatoprak, et al. [17], the total content of bioactive compounds of *P. quercetorum* extracts depended on the solvents used, and the highest content of them was detected in the methanolic extracts (especially from roots). In addition to *P. quercetorum*, several studies have reported significant levels of total bioactive compounds in the extracts of some *Pelargonium* species [18,19,20]. However, the observed results from spectrophotometric assays need to be confirmed by chromatographic techniques including liquid chromatography coupled with different type of detectors, for example, photodiode array or mass spectrometry. 

**Figure 1:**
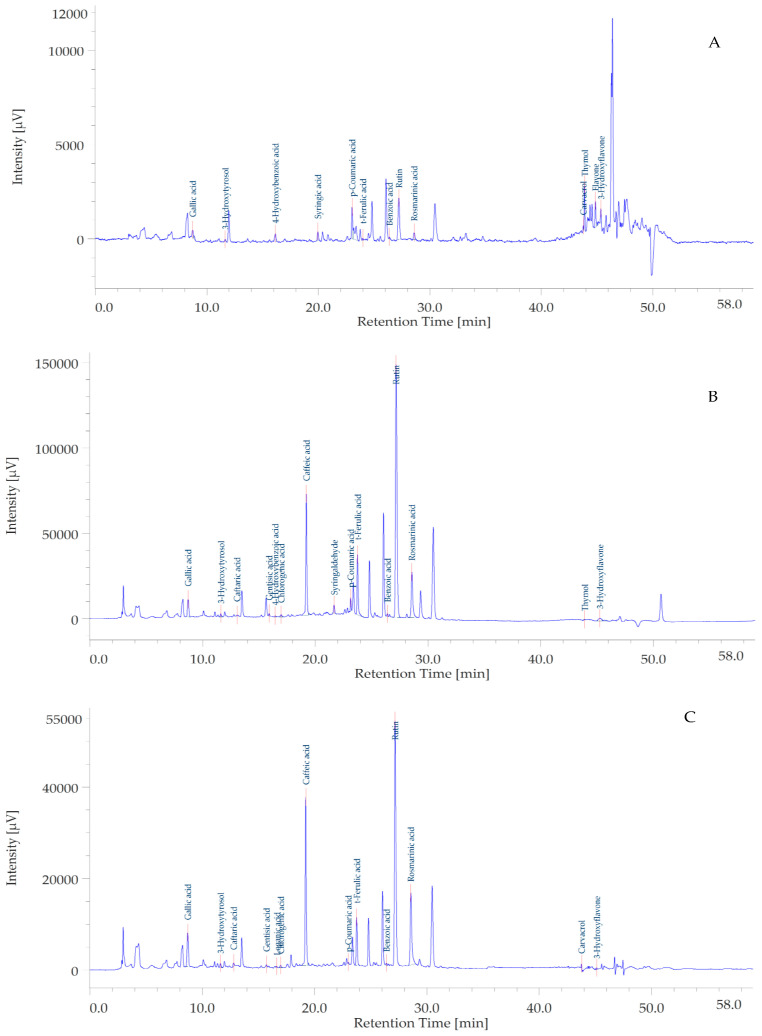
Representative chromatograms of the ethyl acetate (**A**), methanol (**B**), and water (**C**) extracts from the aerial portions of *Pelargonium quercetorum.* The integration of the present chromatograms has been carried out at 254 nm.

Therefore, we conducted a chromatographic analysis of the extracts for the quantitative determination of phenolic compounds, as shown in Table 2 and Figure 1A–C. Specifically, the methanolic extract was the richest in flavonol glycosides and hydroxycinnamic acids, among which rutin, rosmarinic acid, and caffeic acid were the main phytochemicals (Table 2). A similar pattern of phenolic composition, but at lower concentration, was shown by *P. quercetorum* water extract. While the EA extract showed a higher concentration of the phenolic monoterpene thymol.

The content of such phytochemicals could indicate scavenging/reducing and enzyme inhibition properties [21,22], thus substantiating the rationale for exploring innovative health-promoting applications, as described below.

### 2.2. Antioxidant Properties

**Figure 2:**
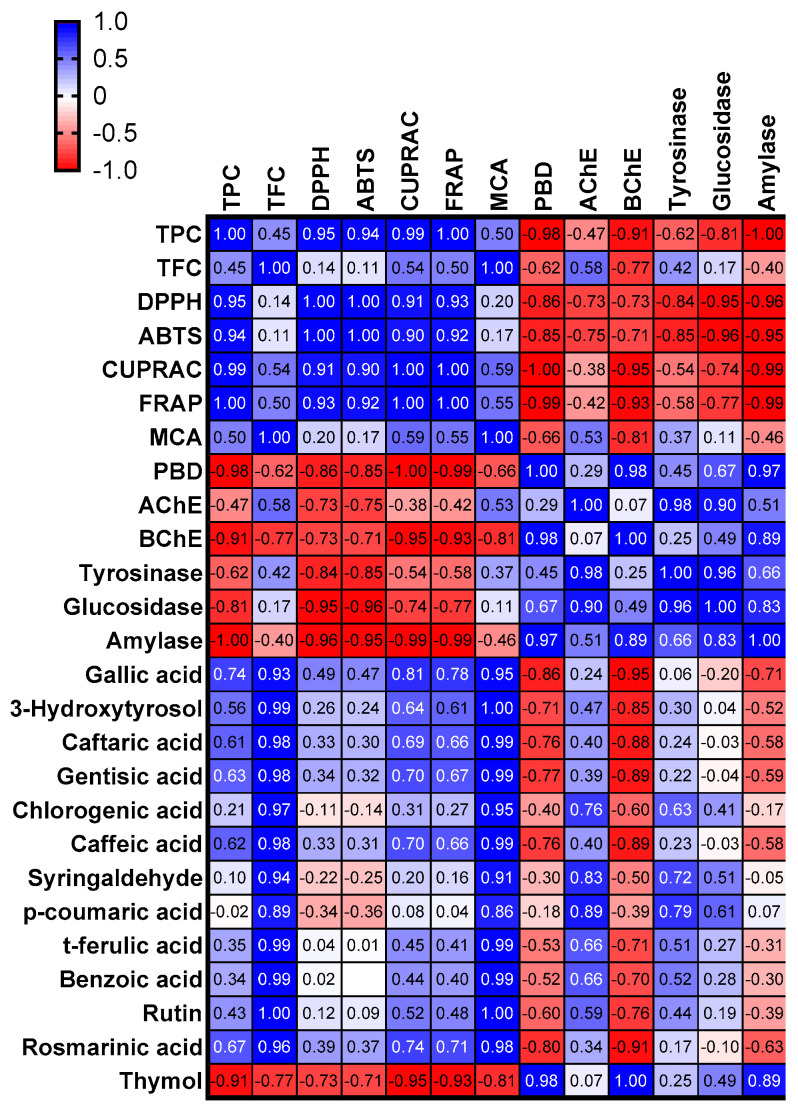
Pearson’s correlations between total/individual bioactive compounds and biological activity assays (*p* < 0.05). TPC: Total phenolic content; TFC: Total flavonoid content; ABTS, 2,2′-azino-bis(3-ethylbenzothiazoline) 6-sulfonic acid; CUPRAC, cupric ion reducing antioxidant capacity; DPPH, 1,1-diphenyl-2-picrylhydrazyl; FRAP, ferric ion reducing antioxidant power; MCA, metal chelating activity; PBD, phosphomolybdenum activity; AChE: Acetylcholinesterase; BChE: Butrylcholinesterase.

According to the latest scientific findings, oxidative stress is an important trigger for the progression of several chronic and degenerative diseases such as cancer, myocardial problems, and diabetes. In particular, increasing the production of free radicals leads to oxidative stress [23,24]. In this sense, antioxidants are the key players in controlling free radical levels. To that end, scientists are looking for new and safe sources of antioxidants, and phytochemicals are believed to be among the most powerful antioxidants. In the present study, we determined antioxidant properties of *P. quercoutum* extracts using different methods such as radical scavenging (2,2′-azino-bis(3-ethylbenzothiazoline) 6-sulfonic acid (ABTS) and 1,1-diphenyl-2-picrylhydrazyl (DPPH)), reducing power (cupric ion reducing antioxidant capacity (CUPRAC) and ferric ion reducing antioxidant power (FRAP)), metal chelating, and phosphomolybdenum assay. The results are summarized in Table 3. In radical scavenging assays (DPPH and ABTS), the best activity was found for the water extract (DPPH: 140.40 mg trolox equivalent (TE)/g extract; ABTS: 212.31 mg TE/g extract), followed by methanol and ethyl acetate extracts. The reducing abilities of antioxidant compounds are very close to their electron-donation ability. Cu^2+^ to Cu and Fe^3+^ to Fe^2+^ conversions were measured using CUPRAC and FRAP assays, respectively. As can be seen from Table 3, the water extract showed the strongest reducing abilities, followed by methanol and ethyl acetate extracts. The results obtained from both free radical scavenger and reducing power assays are consistent with the total phenolic content of the tested extracts. This fact was also confirmed by Pearson’s correlation analysis, and we observed a linear correlation between these parameters (Figure 2). In a literature review, several researchers also reported a strong correlation between total phenolic content and antioxidant properties [25,26,27]. In addition to total phenolic content, some phytochemicals including rosmarinic, ferulic, caffeic, and gallic acids can be attributed to the observed antioxidant properties and are known to be powerful antioxidants [28,29,30,31,32]. In contrast to the radical scavenging ability and reducing power, the order of the tested extracts in the phosphomolybdenum assay was ethyl acetate (1.64 mmol TE/g extract) > methanol (1.54 mmol TE/g extract) > water (1.51 mmol TE/g extract). The phosphomolybdenum assay is known for determining total antioxidant capacity and also non-phenolics could play a role in the assay. Consistent with the presented results, a weak correlation between total phenols and phosphomolybdenum assay were reported by several investigators [33,34]. Finally, we investigated the metal chelating ability of *P. quercetorum* extracts, which is associated with controlling the production of hydroxyl radicals in the Fenton reaction. The methanol extract exhibited the best metal chelating ability with 29.80 mg EDTA equivalent (EDTAE)/g extract, followed by water extract (12.28 mg EDTAE/g extract). However, the ethyl acetate extract showed no metal chelating ability. In a previous study by Karatoprak, Fırat and Koşar [17], the antioxidant properties of *P. quercetorum* aerial parts and root extracts were investigated by DPPH, ABTS, ferric reducing power and metal chelating assays. In their study, the hydroalcoholic root extract in particular showed better potency compared with aerial parts. In addition to *P. quercetorum*, several *Pelargonium* species including *P. hispidum* [35], *P. graveolens* [18], *P. hybrid* [36], and *P. sidoides* [37] were investigated, and their extracts displayed antioxidant properties. With this in mind, the genus *Pelargonium* could be considered a valuable source of natural antioxidants.

### 2.3. Enzyme Inhibitory Properties

Currently, most scientific efforts are focused both on improving the quality of public health and reducing the prevalence of certain diseases such as obesity, diabetes, and cardiovascular diseases [38]. Enzymes play a crucial role at this point and it is known that blocking a specific enzyme is an effective way to treat the abovementioned diseases. In this sense, some enzymes are selected therapeutic targets. For example, acetylcholinesterase is a main target to alleviate the observed symptoms of Alzheimer’s disease [39]. As another example, the carbohydrate-hydrolyzing enzymes, namely α-amylase and α-glucosidase, are checkpoints to control blood glucose level in diabetic patients [40]. For this purpose, several compounds are chemically produced as enzyme inhibitors, but most of them have side effects such as toxicity and gastrointestinal disturbances. At this point, we need to replace the synthetic drugs with natural and safe drugs, and plants are considered a promising in this sense. 

In view of the above facts, we investigated the enzyme inhibitory properties of *P. quercetorum* extracts, and the results are summarized in Table 4. The highest acetylcholinesterase (AChE) inhibitory effect was obtained by the methanol extract with 2.31 mg GALAE/g extract, followed by ethyl acetate and water extracts. However, butyrylcholinesterase (BChE) was only inhibited by the ethyl acetate extract and others were not active on the enzyme. Tyrosinase is the main enzyme involved in the synthesis of melanin, and its inhibition is one of the most effective ways to treat hyperpigmentation problems. As can be seen from Table 4, methanol and ethyl acetate extracts were more active on tyrosinase compared with water. Similar to tyrosinase, the best inhibitory effects on α-amylase and α-glucosidase were recorded in the ethyl acetate and methanol extracts. The water extract exhibited the weakest ability in all enzyme inhibitory assays. As can be seen in Figure 2, the obtained enzyme inhibitory effects could be explained by the presence of some compounds. For example, AChE and tyrosinase inhibitory effects were positively correlated with *p*-coumaric acid, syringaldehyde, and chlorogenic acid, and these compounds have been described by some researchers as anti-Alzheimer and dermatoprotective agents [41,42,43,44,45]. In addition to these components, thymol had a good interaction with anti-diabetic enzymes, namely α-amylase and α-glucosidase. However, the interactions of specialized metabolites such as synergetic and antagonistic effects should not be forgotten. Thus, in future studies, we can suggest that the compounds could be isolated and then tested for their enzyme inhibitory properties. The enzyme inhibition potential of some *Pelargonium* species has been reported in the literature by several researchers. For example, Iancu, et al. [46] investigated the α-amylase and α-glucosidase inhibitory effects of two *Pelargonium* species (*P. hispidum* and *P. grandiflorum*) and found that the methanolic extracts had more potent inhibitory properties than hydroalcholic extracts. In another study conducted by Ahamad and Uthirapathy [47], significant α-amylase and α-glucosidase inhibitory properties were reported for *P. graveolens* essential oil. The IC_50_ value was reported as 10.5 mg/mL in the AChE inhibitory effect of the essential oil of *P. hybrid* [36]. To the best of our knowledge, there are no data on the enzyme inhibitory properties of *P. quercetorum* extracts. Thus, the presented results are the first and they provide new information about the genus *Pelargonium*.

### 2.4. In Vitro Study

**Figure 3:**
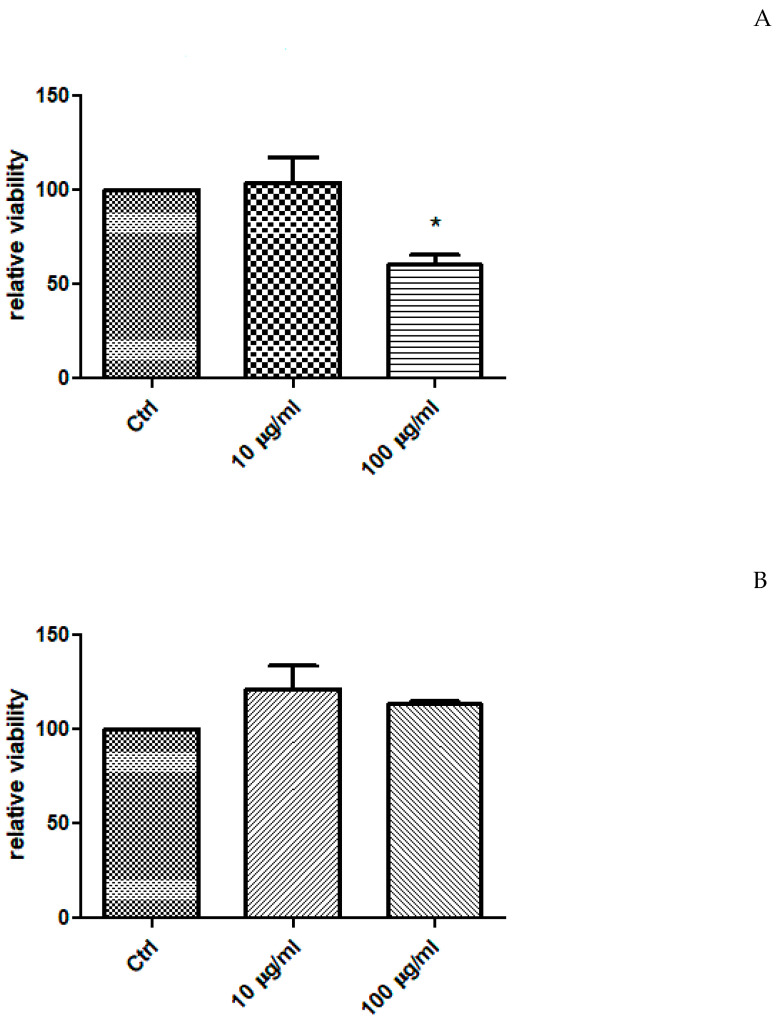

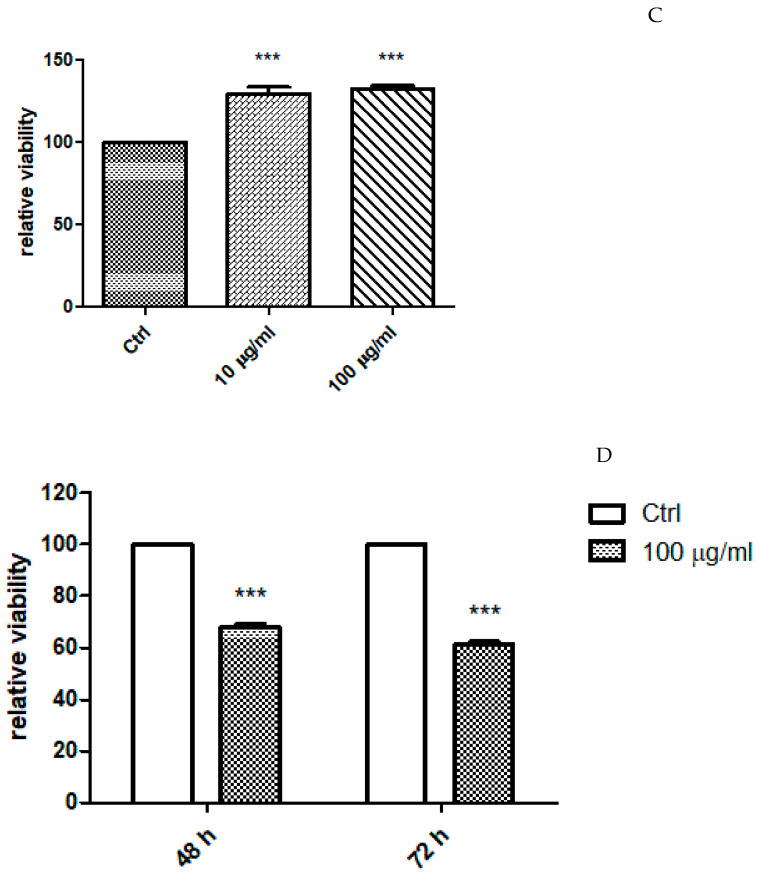
(**A**) Inhibitory effect of the ethyl acetate extract 10–100 µg/mL from the aerial parts of *Pelargonium quercetorum* on human colon cancer HCT116 cell viability. * *p* < 0.05 vs. Ctrl group. (**B**) Null effect of the methanol extract 10–100 µg/mL from the aerial parts of *Pelargonium quercetorum* on human colon cancer HCT116 cell viability. (**C**) Stimulatory effect of the water extract 10–100 µg/mL from the aerial parts of *Pelargonium quercetorum* on human colon cancer HCT116 cell viability. *** *p* < 0.001 vs. Ctrl group. (**D**) Inhibitory effect of the ethyl acetate extract 100 µg/mL from the aerial parts of *Pelargonium quercetorum* on human colon cancer HCT116 cell viability, after 48 and 72 h of exposure to the extract (*** *p* < 0.001).

**Figure 4:**
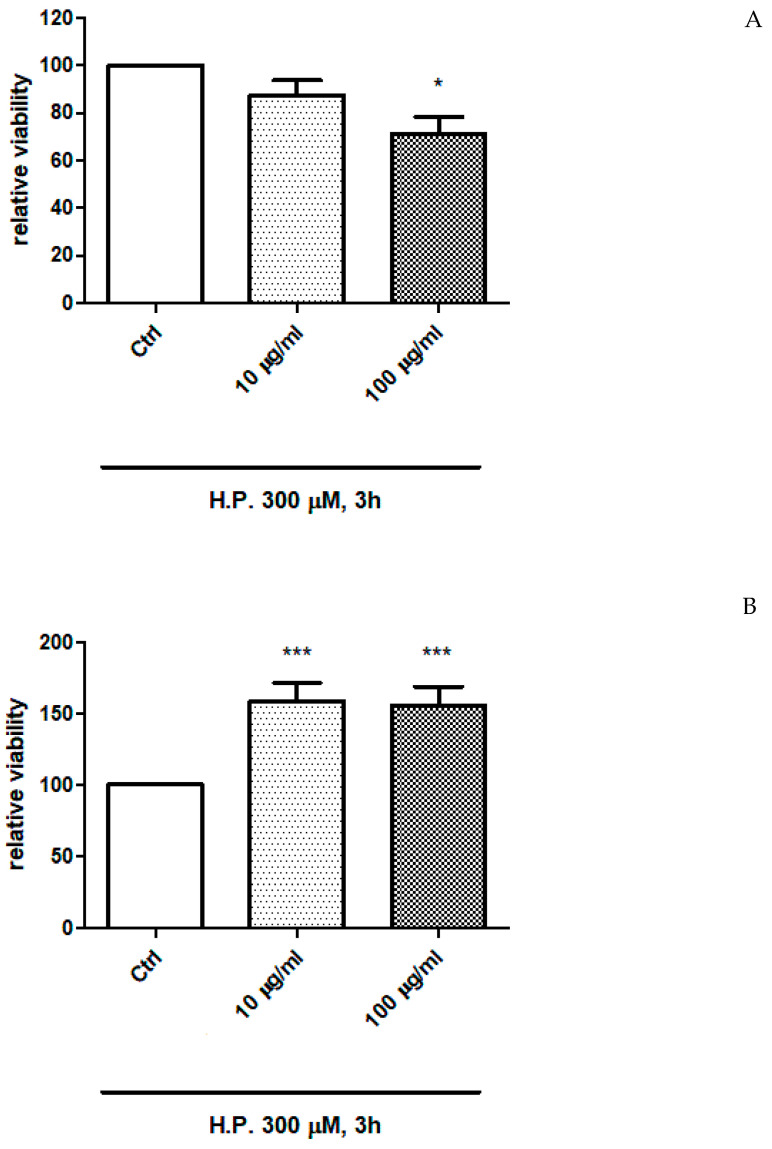

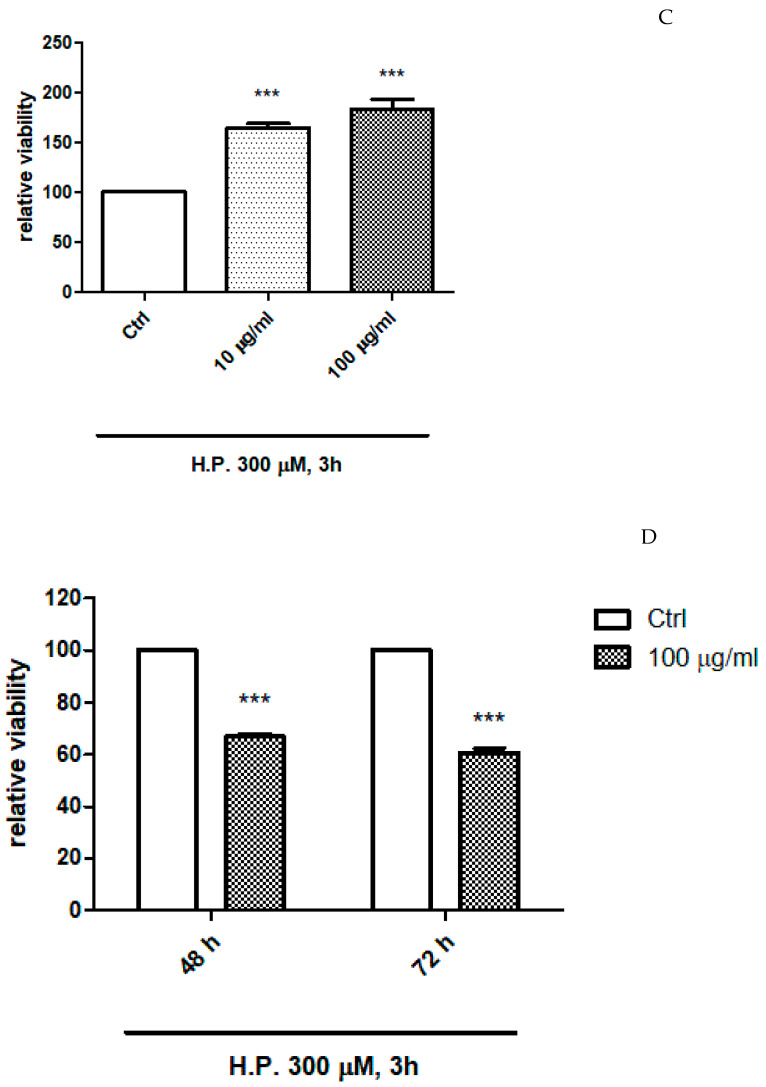
(**A**) Inhibitory effect of the ethyl acetate extract 10–100 µg/mL from the aerial parts of *Pelargonium quercetorum* on the viability of human colon cancer HCT116 cells exposed to hydrogen peroxide (H.P.) 300 µM. * *p* < 0.05 vs. Ctrl group. (**B**) Stimulatory effect of the methanol extract 10–100 µg/mL from the aerial parts of *Pelargonium quercetorum* on the viability of human colon cancer HCT116 cells exposed to hydrogen peroxide (H.P.) 300 µM. *** *p* < 0.001 vs. Ctrl group. (**C**) Stimulatory effect of the water extract 10–100 µg/mL from the aerial parts of *Pelargonium quercetorum* on the viability of human colon cancer HCT116 cells exposed to hydrogen peroxide (H.P.) 300 µM. *** *p* < 0.001 vs. Ctrl group. (**D**) Inhibitory effect of the ethyl acetate extract 100 µg/mL from the aerial parts of *Pelargonium quercetorum* on the viability of human colon cancer HCT116 cells exposed to hydrogen peroxide (H.P.) 300 µM, after 48 and 72 h of exposure to the extract (*** *p* < 0.001).

The extracts of *P. quercetorum* were also investigated in order to evaluate effects on the viability of human colon cancer HCT116 cells. The cells were exposed at different concentrations of the extracts (10–100 µg/mL), finding a significant modulation of cell viability (Figure 3 and Figure 4). Specifically, the ethyl acetate extract was found to be the most effective as cytotoxic agent, with a significant reduction of HCT116 cell viability, at the concentration of 100 µg/mL, also after 48 and 72 h of cell exposure to the extract (Figure 3D and Figure 4D). The effect was more evident in cells not exposed to hydrogen peroxide. While the water and methanol extract stimulated HCT116 cell viability in both basal condition and after challenging with hydrogen peroxide.

**Figure 5:**
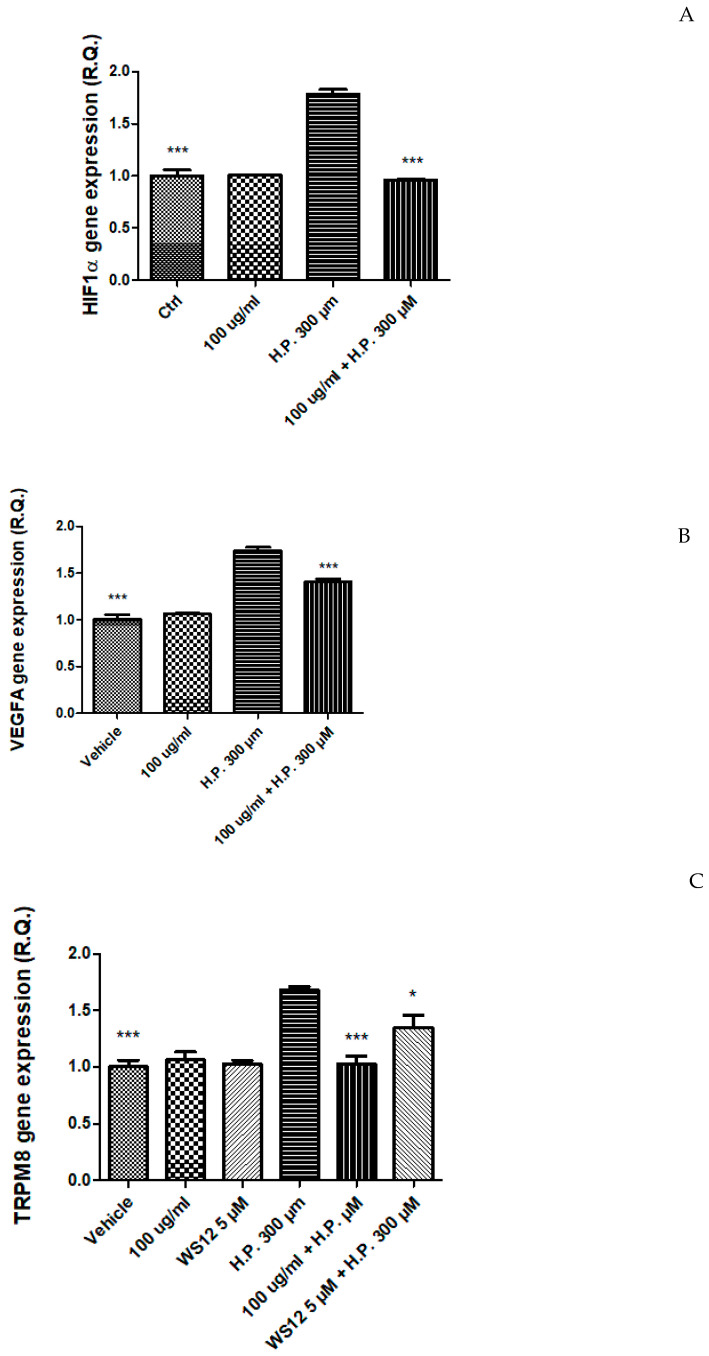
(**A**) Inhibitory effect of the ethyl acetate extract 100 µg/mL from the aerial parts of *Pelargonium quercetorum* on HIF1α gene expression in human colon cancer HCT116 cells exposed to hydrogen peroxide (H.P.) 300 µM. *** *p* < 0.001 vs. H.P. group. (**B**) Inhibitory effect of the ethyl acetate extract 100 µg/mL from the aerial parts of *Pelargonium quercetorum* on VEGFA gene expression in human colon cancer HCT116 cells exposed to hydrogen peroxide (H.P.) 300 µM. *** *p* < 0.001 vs. H.P. group. (**C**) Inhibitory effect of the ethyl acetate extract 100 µg/mL from the aerial parts of *Pelargonium quercetorum* on TRPM8 gene expression in human colon cancer HCT116 cells exposed to hydrogen peroxide (H.P.) 300 µM. *** *p* < 0.001, * *p* < 0.05 vs. Ctrl group.

Therefore, in a second set of experiments, cells were exposed to the EA extract (100 µg/mL) in both basal and oxidative stress conditions [hydrogen peroxide (H.P.) 300 µM], in order to explore the mechanism underlying the cytotoxic effect induced by the extract. In this context, the gene expression of HIF1α and VEGFA, two angiogenic factors deeply involved in the so-called inflammatory-to cancer transition [14] were assayed. Hydrogen peroxide was able to upregulate the gene expression of both HIF1α and VEGFA. This is consistent, albeit partially, with literature [48,49]. The extract was capable to blunt the hydrogen peroxide-induced up-regulation of both factors (Figure 5A,B), and this could be related, albeit partially, to the content in phenolic compounds [50,51]. 

In the same cell model, the gene expression of transient receptor potential (TRP) M8 (TRPM8) was evaluated as well. Indeed, there is evidence of the involvement of TRPM8 receptor in colon cancer [15]. In HCT116 cells, *P. quercetorum* EA extract reduced the hydrogen peroxide-induced upregulation of TRPM8 receptor (Figure 5C), and this effect was analog to the one induced by WS12 ((1R,2S,5R)-2-isopropyl-N-(4-methoxyphenyl)-5-methylcyclohexanecarboxamide), a selective TRPM8 agonist that was used at the concentration of 5 µM, consistent with its putative affinity towards the receptor [52]. 

Regarding the inhibition of the TRPM8 gene expression induced by the extract, the content of thymol could play a pivotal role in mediating this effect [53]. However, we cannot exclude that phenolic compounds present in the extract can mediate, albeit partially, such effect. Indeed, a previous study of ours demonstrated the capability of catechin to reduce the gene expression of TRPM8 [52]. Additionally, flavones, isoflavones, non-prenylated chalcones, and glycocoumarins have been suggested to exert antagonistic effects towards TRPM8 receptor [54]. Therefore, a docking approach was conducted to evaluate the putative affinities of extract phenolics towards TRPM8 receptor, as follows.

### 2.5. Ex Vivo Study

**Figure 6:**
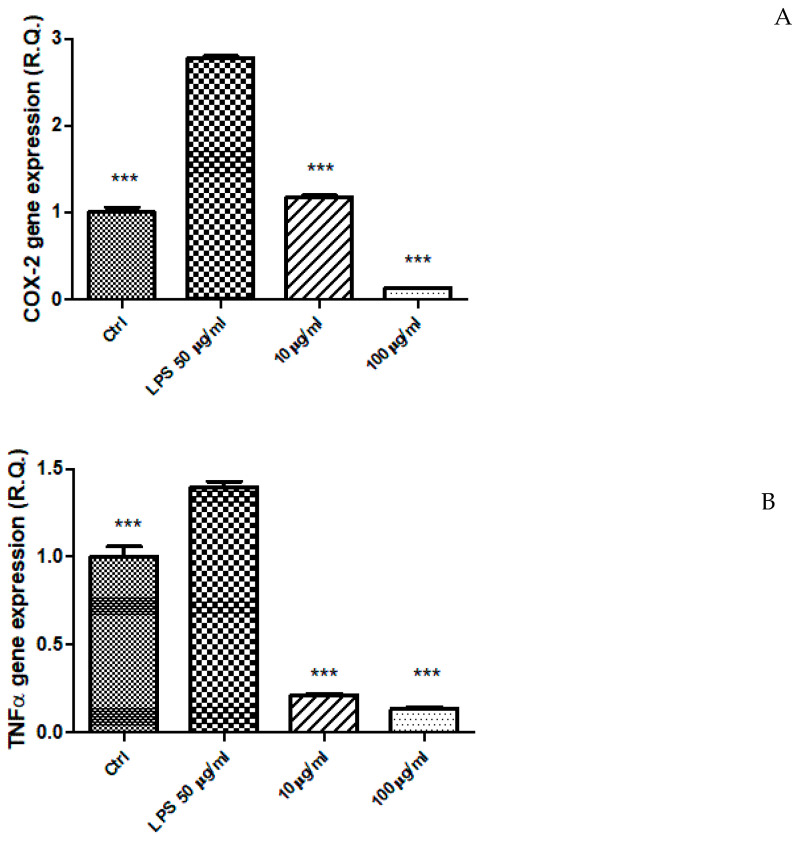
(**A**) Inhibitory effect of the ethyl acetate extract 10–100 µg/mL from the aerial parts of *Pelargonium quercetorum* on COX-2 gene expression in isolated mouse colon specimens exposed to LPS (50 µg/mL). *** *p* < 0.001 vs. LPS group. (**B**) Inhibitory effect of the ethyl acetate extract 10–100 µg/mL from the aerial parts of *Pelargonium quercetorum* on TNFα gene expression in isolated mouse colon specimens exposed to LPS (50 µg/mL). *** *p* < 0.001 vs. LPS group.

The EA extract (10–100 µg/mL) was also tested in an ex vivo experimental model constituted by isolated mouse colon specimens exposed to LPS, in order to induce acute inflammation [8]. The protective effects were evaluated as inhibition of LPS-induced upregulation of the gene expression of pro-inflammatory mediators, namely COX-2 and TNFα (Figure 6A,B). In this context, the extract was effective in reducing the gene expression of both COX-2 and TNFα. This could be partly related to the content in thymol, that was effective in inhibiting the expression of TNFα and COX-2 in LPS-stimulated macrophages [55]. Additionally, our findings of anti-inflammatory effects in the colon are consistent, albeit partially, with the anti-inflammatory effects induced by *P. quercetorum* methanol extract in macrophages exposed to LPS [5]. Being the methanol extract rich in phenolic compounds, particularly rutin, as shown by the present study and by the work of Akkemik et al. [8], it could be hypothesized that different specialized metabolites, including monoterpenes and flavonoids may be involved in the anti-inflammatory effects exerted by *P. quercetorum* extracts.

### 2.6. Molecular Docking

**Figure 7:**
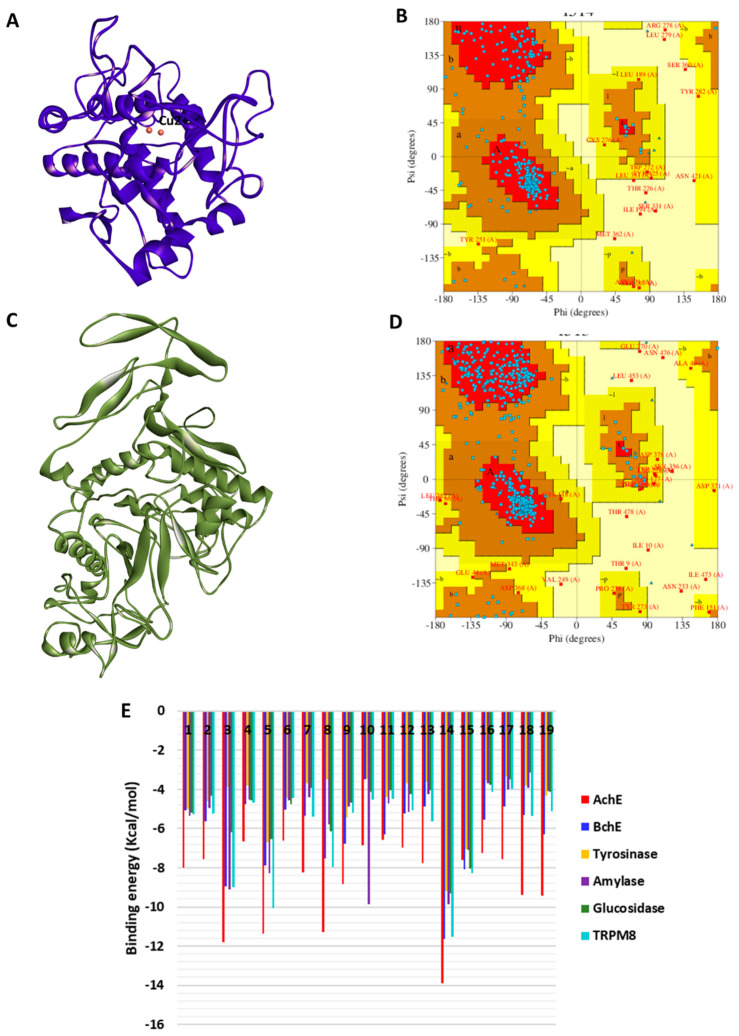
(**A**) Modeled structures of tyrosinase, and (**B**) its Ramachandran plot showing the energetically allowed regions for backbone dihedral angles ψ against φ of amino acid residues. (**C**) Modeled structures of α-glucosidase and (**D**) its Ramachandran plot showing the energetically allowed regions for backbone dihedral angles ψ against φ of amino acid residues. (**E**) Binding energy (docking score) values of the top bioactive compounds from *Pelargonuim quercetorum*. The compounds are displayed based on their serial number in Table 2.

The homology modeling procedure for building human tyrosinase and α-glucosidase models is described in the Section 3. The 3D model of tyrosinase is shown in Figure 7A, and its validation in form of a Ramachandran plot showing energetically allowed regions for backbone dihedral angles ψ against φ of amino acid residues is shown in Figure 7B. The corresponding 3D structure of α-glucosidase is shown in Figure 7C, and its Ramachandran plot, which agreed with those of the known experimental structures, is shown in Figure 7D. The binding energy scores of the bioactive compounds are shown in Figure 7E. All the study compounds exhibited the strongest binding to AChE, and moderate binding to the other enzymes, especially, BChE and α-amylase. 

**Figure 8:**
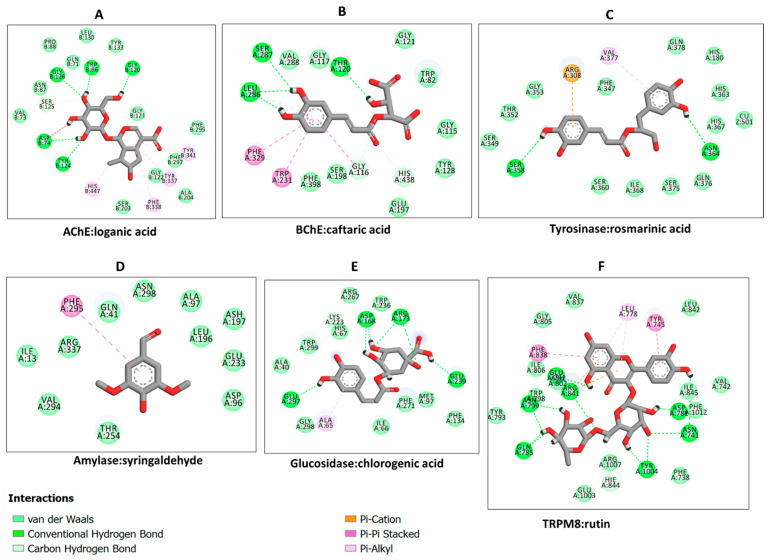
Protein-ligand interactions: (**A**) AChE:loganic acid, (**B**) BChE:caftaric acid, (**C**) tyrosinase:rosmarinic acid, (**D**) α-amylase:syringaldehyde, (**E**) α-glucosidase:chlorogenic acid, and (**F**) TRPM8:rutin.

Hence, the protein–ligand interactions were examined for some selected compounds. Loganic acid and caftaric acid demonstrated a similar binding pattern to AChE and BChE, respectively. Both compounds were completely buried in the active sites of the enzymes and formed a couple of H-bonds and several van der Waals interactions. In addition, loganic acid formed hydrophobic interactions with AChE (Figure 8A), whereas caftaric acid formed π–π stacked interactions with BChE (Figure 8B). Likewise, even though rosmarinic acid shares similar interactions with the enzymes, it uniquely formed π–anion interaction and engaged active site copper metal ion via van der Waals interaction (Figure 8C). On the other hand, syringaldehyde was weakly bound to α-amylase, mainly via van der Waals interactions (Figure 8D). Finally, the interaction between α-glucosidase and chlorogenic acid was demonstrated. Chlorogenic acid formed, mainly, a couple of H-bonds and several van der Waals interactions with residues in the active site of α-glucosidase (Figure 8E). In addition, all the constituent compounds were docked into the binding site of transient receptor potential melastatin member 8 (TRPM8)—an ion channel for cold and menthol sensor. All the compounds were found to bind to the TRPM8, with the strongest binding demonstrated by rutin via multiple interactions with the binding site amino acid residues (Figure 8F); thus, suggesting putative abilities of rutin as regulator of TRPM8 activity. In summary, H-bonds are the major contributor to the protein interaction in all the selected docking complexes, except for that of α-amylase and syringaldehyde, in which van der Waals interactions were predominant. This is due to the multiple hydroxyl groups on the phenolic compounds forming H-bonds with the polar amino acid residues in the active site of the enzymes, corroborating with the binding energy scores. H-bonds are known to contribute greatly to protein–ligand interaction. Other interactions such as hydrophobic and van der Waals interactions reinforce the binding, especially in the case of active sites lined by polar amino acids.

**Figure 9:**
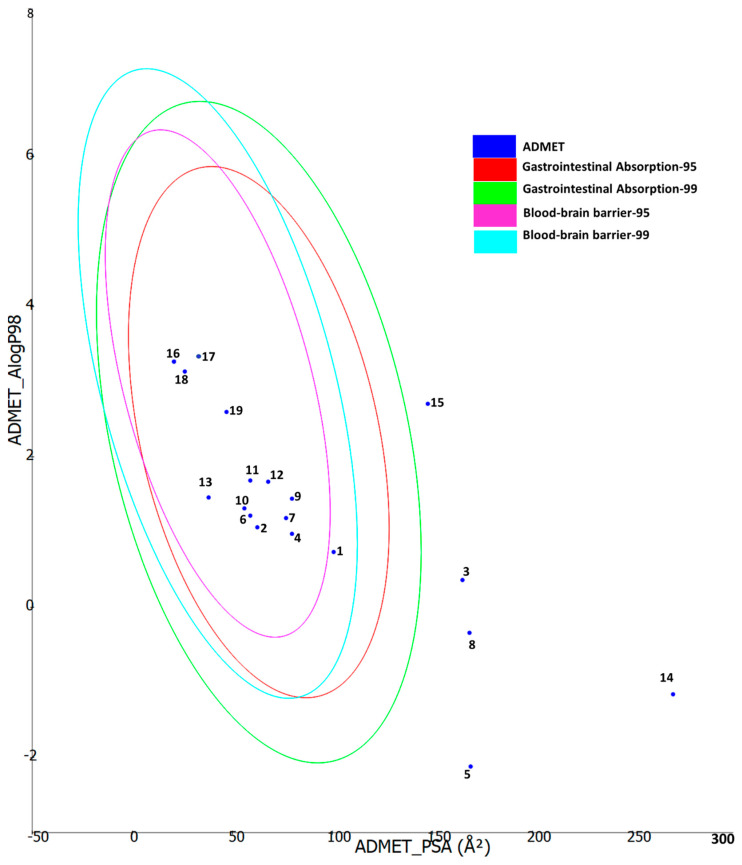
ADMET properties of the bioactive compounds extracted from *Pelargonium quercetorum* predicted using Biovia DS ADMET prediction toolkit. The four ellipses enclose areas where well-absorbed compounds should be found: at 95 and 99% confidence levels for gastrointestinal absorption (red and green), and blood-brain barrier penetration (magenta and aqua). The compounds are shown according to their serial number in Table 2.

Furthermore, ADMET properties of the bioactive constituents were predicted using Biovia DS ADMET prediction toolkit. The four ellipses define the areas where well-absorbed molecules are expected to be located. At 95 and 99% confidence levels of gastrointestinal absorption (red and green), and 95 and 99% blood-brain barrier penetration probability (magenta and aqua). The compounds are shown according to their serial number (Figure 9). Low-molecular-weight compounds associated with low polarity were found to fall in one or more ellipses, suggesting high absorption and high blood-brain barrier penetration possibility, whereas those with high molecular weight and high polarity fell outside the ellipses, suggesting low absorption and low chance of crossing the blood-brain barrier.

## 3. Materials and Methods

### 3.1. Plant Material and Preparation of Extracts

*Pelargonium quercetorum* samples were collected at the flowering season in June 2020 (Şemdinli, Hakkari, Turkey). The plants were identified by one of the authors (Dr. Ugur Cakilcioglu), and voucher specimens (UC-15-21) were deposited at the herbarium of Munzur University, Turkey. The aerial parts (as mixed) were dried in the shade at room temperature for about 7 days, and then ground into a powder using a mill. All of the samples were kept in a dark place.

In this study the extracts were prepared using three solvents (ethyl acetate, methanol, and water). Maceration was employed as the extraction method to obtain ethyl acetate and methanol extracts., The plant materials (5 g) were macerated overnight at room temperature with 100 mL of these solvents. Finally, the solvents were evaporated from the mixtures. To obtain water extracts, the plant materials (10 g) were kept with 200 mL of boiled water for 15 min, and then the extracts were filtered and lyophilized. All extracts were stored at 4 °C until further analysis was required.

### 3.2. Chemicals and Reagents

The chemicals were purchased from Sigma-Aldrich (Darmstadt, Germany). They were ABTS, DPPH, gallic acid, rutin, electric eel acetylcholinesterase (AChE) (type-VI-S, EC 3.1.1.7), horse serum butyrylcholinesterase (BChE) (EC 3.1.1.8), galantamine, acetylthiocholine iodide (ATChI), butyrylthiocholine chloride (BTChI) 5,5-dithio-bis(2-nitrobenzoic) acid (DTNB), tyrosinase (EC1.14.18.1, mushroom), α-glucosidase (EC. 3.2.1.20, from *Saccharomyces cerevisiae*), α-amylase (EC. 3.2.1.1, from porcine pancreas), sodium molybdate, sodium nitrate, sodium carbonate, Folin-Ciocalteu reagent, hydrochloric acid, sodium hydroxide, trolox, ethylenediaminetetraacetate (EDTA), neocuproine, cupric chloride, ammonium acetate, ferric chloride, 2,4,6-Tris(2-pyridyl)-s-triazine (TPTZ), ammonium molybdate, ferrozine, ferrous sulphate hexahydrate, kojic acid, and acarbose. All chemicals were of analytical grade. Chemical standards for HPLC analysis: gallic acid, hydroxytyrosol, caftaric acid, gentisic acid, 4-hydroxybenzoic acid, loganic acid, chlorogenic acid, caffeic acid, syringic acid, syringaldehyde, *p*-coumaric acid, *t*-ferulic acid, benzoic acid, rutin, rosmarinic acid, carvacrol, thymol, flavone, and 3-hydroxyflavone were purchased from Merk Life Science S.r.l. (Milan, Italy).

### 3.3. Determination of Total Polyphenol and Flavonoids Contents

Total phenolic and flavonoid contents were calculated with the Folin-Ciocalteu and AlCl_3_ assays, respectively [56]. Gallic acid equivalents (mg GAEs/g dry extract) and rutin equivalents (mg REs/g dry extract) were used to describe the outcomes of the two tests. Details are reported as Supplementary Materials.

### 3.4. HPLC Determination of Phenolic Compounds

The extract was analyzed for phenol quantitative determination using a reversed-phase HPLC-DAD in gradient elution mode [57]. The separation was conducted within 60 min of the chromatographic run, starting from the following separation conditions: 97% water with 0.1% formic acid, 3% methanol with 0.1% formic acid (Table 5). The separation was performed on an Infinity lab Poroshell 120-SB reverse phase column (C18, 150 × 4.6 mm i.d., 2.7 μm; Agilent, Santa Clara, CA, USA). Column temperature was set at 30 °C. Quantitative determination of phenolic compounds was performed via a DAD detector. The selected wavelengths are reported in Table 2. Quantification was done through 7-point calibration curves, with linearity coefficients (R2) > 0.999, in the concentration range 2–140 μg/mL. All standards were purchased from Sigma Aldrich (Milan, Italy), and have a purity ≥95%. The limits of detection were lower than 1 μg/mL for all assayed analytes. The area under the curve from HPLC chromatograms was used to quantify the analyte concentrations in the extract [57]. 

### 3.5. Antioxidant and Enzyme Inhibitory Assays

The antioxidant and enzyme inhibitory activity of the extracts was assessed according to methods presented previously [58]. Details are also reported as supplementary materials. Data were expressed as mg Trolox equivalents (TE)/g extract in FRAP, CUPRAC, ABTS, and DPPH radical scavenging activity; mg EDTA equivalents (EDTAE)/g extract in the metal chelating ability, mmol TE/g extract in the phosphomolybdenum assay; mg galanthamine equivalents (GALAE)/g extract in AChE and BChE assays; mg kojic acid equivalents (KAE)/g extract in tyrosinase inhibitory assay; and mmol acarbose equivalents (ACAE)/g extract in α-amylase and α-glucosidase assays. 

### 3.6. In Vitro Study

Human colon cancer-derived HCT116 cell were cultured in DMEM (Euroclone) supplemented with 10% (*v*/*v*) heat-inactivated fetal bovine serum and 1% (*v*/*v*) penicillin G/streptomycin in a 75 cm^2^ cell culture flasks. The cultured cells were maintained in a humidified incubator with 5% CO2 at 37 °C. When the confluency reached 80%, a viability test was performed using a 3-(4,5-dimethylthiazol-2-yl)-2,5-diphenyltetrazolium bromide (MTT) test to assess the basal cytotoxicity of the *P. quercetorum* extracts under investigation. For this assay cells were seeded (5 × 10^3^ cells/well) onto flat-bottomed 96-well culture plates and incubated overnight. After 24 h, extracts from the aerial parts of *P. quercetorum* at different concentrations were added, and the plates were incubated for 24, 48, and 72 h. After this time, a total of 10 μL of MTT (5 mg/mL in PBS) was added to each well and incubated for 3 h. The formazan dye formed was solubilized with dimethyl sulfoxide, and the absorbance was recorded at 540 nm as previously described [59]. Effects on cell viability were evaluated in comparison to the vehicle (untreated control group) and expressed as a percentage of the control culture value. 

In a second set of experiments, cells were treated with either vehicle or extract for 24, 48, and 72 h and subsequently post-treated with 300 µM of hydrogen peroxide (H.P.) for 3 h. After this time, cell survival was determined by MTT assay as described above. 

Each condition was run in triplicate, including untreated control and blank cell-free control. 

### 3.7. Ex Vivo Study

Adult C57/BL6 male mice (3-month-old, weight 20–25 g) were housed in plexiglass cages (2–4 animals per cage; 55 cm × 33 cm × 19 cm) and maintained under standard laboratory conditions (21 ± 2 °C; 55 ± 5% humidity) on a 14/10 h light/dark cycle, with ad libitum access to water and food.

Isolated colon specimens were collected form euthanized mice [Project no. F4738.N.5QP] and maintained in a humidified incubator with 5% CO_2_ at 37 °C for 4 h (incubation period), in RPMI buffer with added bacterial LPS (10 µg/mL), as previously described [52]. During the incubation period, the tissues were exposed to the extract (10–100 μg/mL).

### 3.8. RNA Extraction, Reverse Transcription Andreal-Time Reverse Transcription Polymerase Chain Reaction (RT-PCR)

Total RNA was extracted from both HCT116 cells and colon specimens using TRI reagent (Sigma-Aldrich, St. Louis, MO, USA), according to the manufacturer’s protocol, and reverse transcribed using a High Capacity cDNA Reverse Transcription Kit (Thermo Fischer Scientific, Waltman, MA, USA). Gene expression of COX-2, TNF-α, TRPM8, HIF-1α, and VEGF-A were determined by quantitative real-time PCR using TaqMan probe-based chemistry, as previously described [60]. PCR primers and TaqMan probes were purchased from Thermo Fisher Scientific Inc. The Assays-on-Demand Gene Expression Products used for gene expression evaluations in the mouse colon specimens were Mm00478374_m1 for COX-2 gene, Mm00443258_m1 for TNF-α gene, Mm00607939_s1 for β-actin gene. The Assays-on-Demand Gene Expression Products used for gene expression evaluations in the HCT116 cells were: Hs01066596_m1 for TRPM8 gene, Hs00153153_m1 for HIF-1α, Hs00900055_m1 for VEGFA, Hs99999903_m1 for β-actin gene. β-actin was used as the house-keeping gene. The elaboration of data was conducted with the Sequence Detection System (SDS) software version 2.3 (Thermo Fischer Scientific). Relative quantification of gene expression was performed by the comparative 2^−ΔΔCt^ method [61].

### 3.9. Molecular Modelling

AutodockTools program [62] was used to generate docking grid files using the coordinates of the cocrystal ligand in each crystal. The details of the docking procedure were given in our previous works [63,64]. The binding energy of the ligand poses was estimated, and protein–ligand interaction diagrams were generated using the Biovia DS Visualizer.

The following crystal structures of the target enzymes were retrieved from the protein data bank (https://www.rcsb.org/, accessed on 12 January 2023): AChE (PDB ID: 6O52) [65], BChE (PDB ID: 6EQP) [66], tyrosinase (PDB ID: 6QXD) [67] α-amylase (PDB ID: 2QMK) [68], α-glucosidase (PDB ID: 7KBJ) [69], and transient receptor potential melastatin member 8 (TRPM8)(PDB ID: 6NR3) [70]. However, crystal structures of human tyrosinase and α-glucosidase are not available. Therefore, their human sequences: tyrosinase (UniProt ID: P14679) and α-glucosidase (Uniprot ID: P0DUB6) were retrieved from the Uniprot database (https://www.uniprot.org/, accessed on 12 January 2023) and used to build their homology models using the aforementioned PDB structures as templates. ITASSER web-based tool (https://zhanggroup.org/I-TASSER/, accessed on 12 January 2023) [71] was used to build the model and validated using the PROCHECK server (https://www.ebi.ac.uk/thornton-srv/software/PROCHECK/, accessed on 12 January 2023) [72]. 

Protein preparation was performed using an online server “Playmolecule proteinPrepare” (https://playmolecule.com/proteinPrepare/, accessed on 12 January 2023) [73], on which each protein was protonated using the predicted pKa of the titratable residues at physiological pH of 7.4. The 3D structure of each ligand was downloaded from the PubChem database (https://pubchem.ncbi.nlm.nih.gov/, accessed on 12 January 2023), and an optimized conformation was generated using Frog2 [74].

### 3.10. Statistical Analysis

In the antioxidant and enzyme inhibitory assays, the values are expressed as mean ± SD of three parallel experiments. To determine the differences between tested extracts in terms of antioxidant and enzyme inhibitory capacities, one-way analysis of variance (ANOVA) with Tukey test was performed. The statistical analysis was performed using XlStat 16.0 software.

In ex vivo and in vitro studies, the software GraphPad Prism version 5.01 (Graphpad Software Inc., San Diego, CA, USA) was used to perform data analysis. Means ± SEM were determined for each experimental group and analyzed by ANOVA, followed by Newman-Keuls multiple comparison post hoc test. The limit of statistically significant differences between mean values was set at p-value < 0.05. The number of animals randomized for each experimental group was calculated based on the “Resource Equation” N = (E + T)/T (10 ≤ E ≤ 20).

## 4. Conclusions

The present study explored the composition and biological properties of extracts of different polarity from the aerial parts of *P. quercetorum,* a plant traditionally used by the local population in the Hakkari region of Turkey. The extracts showed a different qualitative and quantitative content of phytochemicals, with water and methanol extracts being richer in total phenols and flavonoids, especially rutin and hydroxycinnamic acids, namely caffeic acid and rosmarinic acid. This could explain, albeit partially, the higher antioxidant effects showed by methanol and water extracts, compared with ethyl acetate extract. By contrast, the ethyl acetate was more effective as cytotoxic agent against colon cancer cells, and this could be related, albeit partially, to the content of thymol and to its ability to downregulate the gene expression of TRPM8, an endovanilloid receptor possibly involved in colon carcinogenesis. Intriguingly, docking runs also suggest a high putative affinity of rutin towards TRPM8. This could suggest a potential involvement of rutin in mediating, albeit partially, the cytotoxic effects induced by the ethyl acetate extract of *P. quercetorum,* in human colon cancer HCT116 cells. Finally, the ethyl acetate extract reduced the gene expression of COX-2 and TNFα gene expression, in isolated colon specimens challenged with LPS; thus, supporting further in vitro and vivo studies for investigating protective effects against gut inflammatory diseases. Additionally, further toxicological investigations are needed for defining the limits of biocompatibility in vivo.

## Figures and Tables

**Table 1:** Total phenolic (TPC) and flavonoid content (TFC) of the tested extracts.

Solvents	TPC (mg GAE/g Extract)	TFC (mg RE/g Extract)
Ethyl acetate	21.94 ± 0.72 ^c^	2.78 ± 0.58 ^c^
Methanol	45.77 ± 1.02 ^b^	40.12 ± 0.20 ^a^
Water	62.52 ± 0.43 ^a^	16.15 ± 0.17 ^b^

Values are reported as mean ± SD of three parallel experiments. TPC: Total phenolic content; TFC: Total flavonoid content; GAE: Gallic acid equivalent; RE: Rutin equivalent. Different letters indicate significant differences in the tested extracts (*p* < 0.05).

**Table 2:** Content in specialized metabolites (µg/mg) of the tested *P. quercetorum* extracts. All identified phytochemicals have been identified through comparison with pure standards. Most of them have been quantified in at least one extract. Quantitative determination of the compounds was performed via DAD detector at 254–340 nm wavelength.

Components		Chemical Class	EA	MEOH	Water	Retention Time (Min)	Wavelenghts
1	Gallic acid	Benzoic acids	nq	12.55 ± 0.02	8.53 ± 0.03	8.723	271
2	Hydroxytyrosol	Phenylethanoids	nq	13.78 ± 0.03	6.18 ± 0.14	11.620	280
3	Caftaric acid	Hydroxycinnamic acids	nq	4.70 ± 0.15	3.20 ± 0.04	13.060	310
4	Gentisic acid	Benzoic acids	nq	5.03 ± 0.02	1.70 ± 0.21	15.907	325
5	4-Hydroxybenzoic acid	Benzoic acids	nq	nq	nq	16.450	256
6	Loganic acid	Iridoids	nq	nq	nq	16.637	
7	Chlorogenic acid	Hydroxycinnamic acids	nq	1.70 ± 0.20	0.40 ± 0.03	16.960	325
8	Caffeic acid	Hydroxycinnamic acids	nq	51.75 ± 1.20	27.18 ± 0.44	19.207	325
9	Syringic acid	Benzoic acids	nq	nq	nq	19.960	274
10	Syringaldehyde	Benzoic aldehydes	nq	28.35 ± 0.25	nq	21.667	310
11	*p*-Coumaric acid	Hydroxycinnamic acids	1.43 ± 0.18	13.53 ± 0.35	nq	23.140	310
12	*t*-Ferulic acid	Hydroxycinnamic acids	nq	33.23 ± 0.21	8.80 ± 0.09	23.747	315
13	Benzoic acid	Benzoic acids	1.05 ± 0.02	8.35 ± 0.26	3.18 ± 0.05	26.400	275
14	Rutin	Flavonol glycosides	1.05 ± 0.01	190.33 ± 0.92	63.78 ± 0.98	27.170	254
15	Rosmarinic acid	Hydroxycinnamic acids	nq	45.28 ± 0.44	27.13 ± 0.38	28.570	325
16	Carvacrol	Phenolic monoterpenes	nq	nq	nq	43.757	275
17	Thymol	Phenolic monoterpenes	15.68 ± 0.02	nq	nq	43.873	275
18	Flavone	Flavones	nq	nq	nq	44.840	340
19	3-Hydroxyflavone	Flavones	nq	nq	nq	45.327	340

nq: not quantified.

**Table 3:** Antioxidant properties of the tested extracts.

Solvents	DPPH (mg TE/g Extract)	ABTS (mg TE/g Extract)	CUPRAC (mg TE/g Extract)	FRAP (mg TE/g Extract)	MCA (mg EDTAE/g Extract)	PBD (mmol TE/g Extract)
Ethyl acetate	9.28 ± 0.52 ^c^	27.17 ± 0.04 ^c^	96.07 ± 1.11 ^c^	32.65 ± 0.70 ^c^	na	1.64 ± 0.04 ^a^
Methanol	48.80 ± 0.01 ^b^	78.59 ± 0.10 ^b^	172.02 ± 6.72 ^b^	102.48 ± 6.20 ^b^	29.80 ± 0.79 ^a^	1.54 ± 0.07 ^b^
Water	140.40 ± 1.16 ^a^	212.31 ± 8.88 ^a^	207.83 ± 1.36 ^a^	142.01 ± 1.08 ^a^	12.28 ± 0.23 ^b^	1.51 ± 0.01 ^b^

Values are reported as mean ± SD of three parallel measurements. TE: Trolox equivalent; EDTAE: EDTA equivalent; na: not active. Different letters indicate significant differences in the tested extracts (*p* < 0.05). ABTS, 2,2′-azino-bis(3-ethylbenzothiazoline) 6-sulfonic acid; CUPRAC, cupric ion reducing antioxidant capacity; DPPH, 1,1-diphenyl-2-picrylhydrazyl; FRAP, ferric ion reducing antioxidant power; MCA, metal chelating activity; PBD, phosphomolybdenum activity.

**Table 4:** Enzyme inhibitory of the tested extracts.

Solvents	AChE (mg GALAE/g Extract)	BChE (mg GALAE/g Extract)	Tyrosinase (mg KAE/g Extract)	α-Amylase (mmol ACAE/g Extract)	α-Glucosidase (mmol ACAE/g Extract)
Ethyl acetate	1.63 ± 0.11 ^b^	2.59 ± 0.04	22.68 ± 1.26 ^b^	0.91 ± 0.02 ^a^	1.37 ± 0.07 ^a^
Methanol	2.31 ± 0.29 ^a^	na	31.24 ± 0.49 ^a^	0.45 ± 0.03 ^b^	1.38 ± 0.02 ^a^
Water	0.76 ± 0.08 ^c^	na	0.63 ± 0.11 ^c^	0.07 ± 0.01 ^c^	0.40 ± 0.01 ^b^

Values are reported as mean ± SD of three parallel measurements. GALAE: Galantamine; KAE: Kojic acid; ACAE: Acarbose equivalent; na: not active. Different letters indicate significant differences in the tested extracts (*p* < 0.05). AChE: Acetylcholinesterase; BChE: Butrylcholinesterase.

**Table 5:** Gradient Elution Conditions.

Time (Min.)	Compasition A% (Water + Formic Acid 0.1%)	Compasition B% (Methanol + Formic Acid 0.1%)	Flow (mL/Min)
1.00	97.0	3.0	0.600
5.00	77.0	23.0	0.600
12.00	73.0	27.0	0.600
18.00	57.0	43.0	0.600
25.00	52.0	48.0	0.600
32.00	50.0	50.0	0.600
34.00	50.0	50.0	0.600
37.00	35.0	65.0	0.600
40.00	5.0	95.0	0.600
47.00	5.0	95.0	0.600
48.00	97.0	3.0	0.600
60.00	97.0	3.0	0.600

## Data Availability

The data that support the findings of this study are available from the corresponding author.

## Supplementary Materials

The following supporting information can be downloaded at: https://www.mdpi.com/article/10.3390/plants12051132/s1, Assays for Total Phenolic and Flavonoid Contents; Determination of Antioxidant and Enzyme Inhibitory Effects. Refs. [58,75] cited in Supplementary Materials.
